# Risk of Death Influences Regional Variation in Intensive Care Unit Admission Rates among the Elderly in the United States

**DOI:** 10.1371/journal.pone.0166933

**Published:** 2016-11-29

**Authors:** Colin R. Cooke

**Affiliations:** 1 Division of Pulmonary and Critical Care Medicine, Department of Medicine, University of Michigan, Ann Arbor Michigan, United States of America; 2 Center for Healthcare Outcomes & Policy, Institute for Healthcare Policy and Innovation, University of Michigan, Ann Arbor, Michigan, United States of America; 3 Michigan Center for Integrative Research in Critical Care, University of Michigan, Ann Arbor, Michigan, United States of America; Yokohama City University, JAPAN

## Abstract

**Rationale:**

The extent to which geographic variability in ICU admission across the United States is driven by patients with lower risk of death is unknown.

**Objectives:**

To determine whether patients at low to moderate risk of death contribute to geographic variation in ICU admission.

**Methods:**

Retrospective cohort of hospitalizations among Medicare beneficiaries (age > 64 years) admitted for ten common medical and surgical diagnoses (2004 to 2009). We examined population-adjusted rates of ICU admission per 100 hospitalizations in 304 health referral regions (HRR), and estimated the relative risk of ICU admission across strata of regional ICU and risk of death, adjusted for patient and regional characteristics.

**Measurement and Main Results:**

ICU admission rates varied nearly two-fold across HRR quartiles (quartile 1 to 4: 13.6, 17.3, 20.0, and 25.2 per 100 hospitalizations, respectively). Observed mortality for patients in regions (quartile 4) with the greatest ICU use was 17% compared to 21% in regions with lowest ICU use (quartile 1) (p<0.001). After adjusting for patient and regional characteristics, including regional differences in ICU, skilled nursing, and long-term acute care bed capacity, individuals’ risk of death modified the relationship between regional ICU use and an individual’s risk of ICU admission (p for interaction<0.001). Region was least important in predicting ICU admission among patients with high (quartile 4) risk of death (RR 1.27, 95% CI 1.22–1.31, for high versus low ICU use regions), and most important for patients with moderate (quartile 2; RR 1.63, 95% CI 1.53–1.72, quartile 3; RR 1.56 95% CI 1.47–1.65) and low (quartile 1) risk of death (RR 1.50, 95% CI 1.41–1.59).

**Conclusions:**

There is wide variation in in ICU use by geography, independent of ICU beds and physician supply, for patients with low and moderate risks of death.

## Introduction

For over three decades researchers and policy experts have examined how marked variation in the use of the intensive care unit (ICU) may reflect waste and inefficiency in healthcare[1–4]. Studies consistently conclude there are several-fold differences in rates of ICU admission and the total number of ICU days used for otherwise similar hospitalized patients, particularly those at the end of life, across both hospitals and geographic regions[3, 4]. This variability in ICU use is not consistently associated with outcome differences, prompting policymakers to implement programs such as accountable care organizations to address its underlying causes and ultimately reduce unnecessary healthcare spending.

The causes of such variation in ICU use, however, are largely unknown. Many researchers have examined the problem of variation in ICU use through a lens of care of patients who are severely ill and near the end of life[5, 6]. Advantages of focusing on this population include that most Americans view the ICU as an unwanted location to die, and care of patients at the end of life accounts for a disproportionate share of healthcare spending[7, 8]. Some researchers conclude from this body of work that the problem of inefficiency in use of the ICU is solely attributable to the care of patients who are dying. Yet this perception neglects the 75% of healthcare spending that occurs among those who are not at the end of life, a group in which spending is growing at a faster rate[8]. Moreover, recent studies suggest that up to of 50% of patients admitted to ICUs do not require life support or have very low predicted risks of death, suggesting they may not benefit from ICU services[9]. Together, these studies suggest that our current understanding of the causes of variation in ICU use is incomplete because it fails to account for the contribution of patients with low anticipated short-term mortality to variability in ICU use. National efforts to improve efficiency in use of the ICU by targeting patients at the end of life may ultimately fail if this population accounts for a minority of the variation in how ICUs are used.

We sought to characterize how an individual’s risk of death contributes to and modifies their likelihood of ICU admission across geographic regions. Specifically, we examined patients hospitalized for several common medical and surgical diagnoses in the Medicare population to determine how risk of death modifies the impact of region on an individual’s likelihood of ICU admission. We hypothesized that individuals with a low risk of death would disproportionately contribute to geographic variation in use of the ICU.

## Methods

### Study cohort and data sources

We used Medicare Provider Analysis and Review and Beneficiary Summary files to identify all acute care hospitalizations for one of several conditions among fee-for-service Medicare beneficiaries aged 65 years or older between 2004–2009. We included patients admitted with acute myocardial infarction, congestive heart failure, chronic obstructive pulmonary disease, pneumonia, gastrointestinal hemorrhage, acute renal failure, ischemic stroke, colectomy, hip fracture surgery, and non-cervical spine fusion. We selected these conditions because they are among the most common reasons for admissions in the Medicare population, are frequently, though not universally, admitted to an ICU, and have in hospital mortality rates of greater than 1%. We identified these conditions using standard ICD-9 codes present in the primary discharge diagnosis field (see S1 Table).

We limited our analysis to acute care hospitals that billed for ICU care to Medicare. We linked all hospitalizations to the Healthcare Cost Report and Information System and the American Hospital Association Annual Survey to provide hospital characteristics. We used the Dartmouth Atlas of Healthcare and annual US census estimates to provide characteristics and population denominators for calculation of available resources (e.g. ICU beds per capita) in the healthcare region where each individual resided based on ZIP codes[10]. The geographic unit for estimating ICU admission rates and available resources were the 304 (excluding Alaska and Hawaii) healthcare referral regions (HRR) as defined by the Dartmouth Atlas.

We chose to examine variation across HRRs, as opposed to hospitals, for three primary reasons. First, there is heterogeneity in the patient populations that seek care at different hospitals, much of which is unmeasured in Medicare claims data. Moreover, heterogeneity is greater across hospitals than that present across large geographic regions[11]. Thus, our use of HRR averages out some (but not all) unmeasured differences in patients across hospitals. Second, a sizeable proportion of critically ill patients are transferred between hospitals, but very few are transferred across regions[12, 13]. Finally, many of the systems level factors (e.g. number of intensivists) that were included in our analysis are best, if not *only* assessed at the population level.

### Determining population-based ICU admission rates

To determine the regional rate of ICU admission we first identified hospitalizations involving an ICU stay using revenue center codes, excluding intermediate ICU admissions, as previously defined[14]. We then assigned all ICU admissions to an HRR based upon the individual’s ZIP code, summed ICU admissions within each HRR, stratified by age, sex, race and annual US Census-based socioeconomic status (SES)[15, 16], and divided by the eligible population of strata-specific hospitalizations. SES was derived using a composite measure of six Census variables representing the dimensions of wealth and income (the median household income; median value of housing units; and the percentage of households receiving interest, dividend, or net rental income), education (the percentage of adults 25 years of age or older who had completed high school and the percentage of adults 25 years of age or older who had completed college), and occupation (the percentage of employed persons 16 years of age or older in executive, managerial, or professional specialty occupations) 1. We then age, sex, race, and SES standardized ICU admission rates—herein referred to as adjusted ICU admission rates—in the HRRs to the entire population included in the study. This effectively adjusts ICU admission rates in each HRR for age, sex, race and socioeconomic differences across HRRs. We then divided HRRs into quartiles of adjusted ICU admission rates and mapped the distribution of HRR quartiles across the US.

### Estimating individual risk of death

To estimate the risk of death for ICU admissions, we generated a multivariable logistic regression model where hospitalization was the unit of analysis, 30-day mortality was the outcome, and included age, sex, race, SES, Elixhauser comorbidities[17], qualifying diagnosis, number of organ failures[18], and several secondary diagnoses or procedures associated with receipt of critical care services in the model[19]. The mortality model included variables thought to plausibly predict 30-day mortality that available in the hospitalization file. No variables were selected based upon statistical significance, nor were interaction terms evaluated. Discrimination of the model was assessed with the area under the receiver operating characteristic curve (AUC). Calibration was assessed using the Hosmer-Lemeshow statistic. We then generated model-based probabilities of death for each hospitalization and divided the distribution of probabilities for all ICU patients into quartiles.

### Statistical analysis

We calculated summary statistics for regional characteristics and characteristics of patients admitted to the ICU across regional quartiles of adjusted ICU admission rates using percentages, means (standard deviations [SDs]), and medians (interquartile ranges [IQRs]).

The primary goal of our analysis was to determine whether the relationship between regional ICU admission rates and an individual’s likelihood of being admitted to the ICU differed by the individual’s risk of death. To achieve this goal, we first plotted the proportion of individuals in each quartile of regional adjusted ICU admission rates that had a low, moderate-low, moderate-high, and high risk of death (as defined by quartiles of predicted 30-day mortality). We then entered the quartile of regional adjusted ICU admission rate into a logistic regression model as a categorical variable. We interacted this variable with the patient-level quartile of risk of death. Patient-level ICU admission was the outcome. We then tested the significance of the interaction term and generated predictive margins for the model and estimated relative risks from the posterior predictions using stata’s nlcom command. Finally, we plotted these relative risks of ICU admission to allow comparisons across quartile of regional ICU admission rate and individual patient risk of death.

The model was adjusted for several aggregate patient and regional characteristics for the HRR: mean age, proportion female, proportion black race, mean SES, fraction of individuals with > 3 organ failures, fraction of individuals with > 3 comorbid diagnoses, mean annual ICU bed occupancy, per capita skilled nursing facility (SNF) beds, ICU, and long term acute care (LTAC) beds, regional population density, per capita intensivists, per capita specialists, per capita hospitalizations, market competition (Herfindahl–Hirschman Index[20]), and year. Per-capita measures of bed supply were normalized to the entire resident population, not just Medicare beneficiaries. Non-independence of outcomes within a region was accounted for in the model using generalized estimating equations using HRR as the clustering variable with robust variance estimates.

Data management and analysis were performed using SAS 9.3 (SAS Institute, Cary, NC) and Stata 14 (Statacorp, College Station, TX). We conducted the study under a data-use agreement with the Centers for Medicare and Medicaid Services and received approval from the institutional review board of the University of Michigan (HUM00053488) under a waiver of informed consent. Data was anonymized and de-identified prior to analysis.

## Results

**Fig 1:**
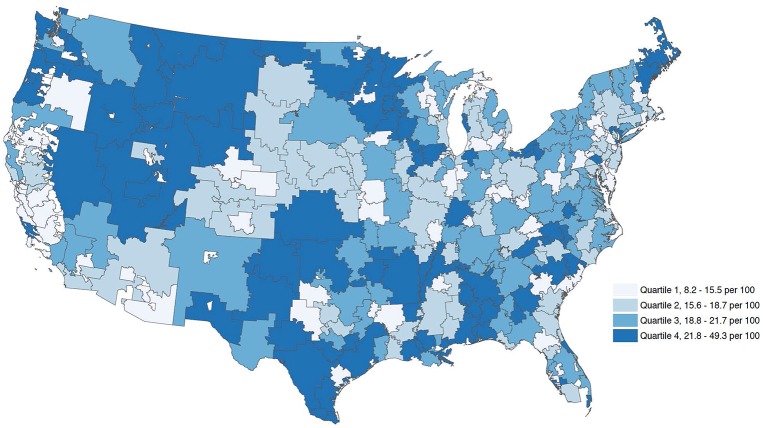
Variation in age, sex, race and socioeconomic status standardized ICU admission rates (per 100 hospitalization) across health referral regions the United States. Socioeconomic status was derived using a composite measure of six Census variables representing the dimensions of wealth and income (the median household income; median value of housing units; and the percentage of households receiving interest, dividend, or net rental income), education (the percentage of adults 25 years of age or older who had completed high school and the percentage of adults 25 years of age or older who had completed college), and occupation (the percentage of employed persons 16 years of age or older in executive, managerial, or professional specialty occupations).

During the study period, there were 15,047,516 hospitalizations with a qualifying condition admitted to acute care hospitals with an ICU. Twenty percent (n = 3,003,592) were admitted to the ICU. Age, sex, race, and SES-standardized rates of ICU admission per 100 hospitalizations varied dramatically across HRRs, from 8.2% to 49.3%. When divided into HRR quartiles, median ICU admission rates were 13.6%, 17.3%, 20.0% and 25.2% of hospitalizations for quartiles one through four, respectively. Geographic variation in the use of the ICU is shown in Fig 1. An interactive version of Fig 1 is available at https://s3-us-west-2.amazonaws.com/colinrcooke/visualization/incidencemap.html

### Characteristics of ICU admissions and regions

ICU patients in regions with the highest quartile of ICU admissions were more often black, had lower SES, had more comorbid diagnoses, but fewer organ failures during their hospital stay (Table 1). Mortality was 20.7% in the lowest quartile of ICU admission and declined across quartiles to 16.6% in the highest quartile of ICU admission. Concordance between observed and predicted mortality across quartiles was high. There were also small but statistically significant differences in age, sex, and case-mix across regions. Regions with the highest ICU admission rates had greater per-capita ICU beds, long-term acute care beds, and skilled nursing beds (Table 2). However, skilled nursing beds were greatest in quartile 3. High ICU use regions also had the fewest available per-capita intensivists and specialists.

**Table 1:** Characteristics of patients across HRR quartiles of age, sex, race, standardized ICU utilization.

Characteristics of patients by HRR	Median (range) ICU admissions per 100 hospitalizations in HRR				P value
	Quartile 1	Quartile 2	Quartile 3	Quartile 4	
	13.6% (8.1–15.5%)	17.3% (15.6–18.7)	20.0% (18.7–21.7)	25.2% (21.8–49.3)	
Number of ICU admissions(total)	373,146	813,939	942,909	873,598	
Age(%)					
65–74 yrs	34.7	35.4	35.2	35.0	<0.001
75–84 yrs	40.6	40.6	40.3	40.4	<0.001
85+ yrs	24.7	24.0	24.5	24.6`	<0.001
Female(%)	51.8	51.9	51.8	52.0	0.02
Race(%)					
Black	8.1	10.7	8.5	9.4	<0.001
Non-Black	91.9	89.3	91.5	90.6	
SES(%)					
1 (low)	11.9	12.6	14.9	18.5	<0.001
2	20.8	23.1	27.7	29.5	<0.001
3 (high)	67.3	64.3	57.5	52.0	<0.001
Diagnosis (%)					
CHF	22.8	23.1	22.6	22.6	<0.001
Pneumonia	19.3	19.5	19.4	19.7	<0.001
COPD	11.3	11.6	11.7	11.2	<0.001
AMI	10.9	10.7	11.6	11.1	<0.001
Ischemic stroke	8.5	8.6	8.4	8.6	<0.001
Hip fracture repair	8.0	7.8	7.9	8.2	<0.001
Renal failure	6.7	6.8	6.6	6.2	<0.001
GI bleed	6.4	6.3	6.2	6.3	<0.001
Colectomy	3.8	3.6	3.5	3.6	<0.001
Non-cervical spine fusion	2.3	2.1	2.1	2.5	<0.001
# of Comorbidities(%)					
0	7.2	6.8	6.8	6.6	<0.001
1	21.3	21.9	21.1	20.8	<0.001
2	29.8	29.7	29.6	29.3	<0.001
3+	41.2	42.2	42.5	43.4	<0.001
# of Organ Failures (%)					
0	58.4	59.9	62.5	67.1	<0.001
1	28.3	27.9	26.6	24.2	<0.001
2	10.2	9.4	8.5	6.8	<0.001
3+	3.1	2.8	2.4	1.8	<0.001
Actual 30-day Mortality (%)	20.7	19.5	18.9	16.6	<0.001
Predicted 30-day Mortality (%)	21.0	19.6	18.4	16.3	<0.001

AMI, acute myocardial infarction, CHF, congestive heart failure; COPD, chronic obstructive pulmonary disease; GI, gastrointestinal; HRR, health referral region; ICU, intensive care unit; SES, socioeconomic status.

**Table 2:** Availability of regional resources.

Characteristics of HRR	Median (range) ICU admissions per 100 hospitalizations in HRR				P value
	Quartile 1	Quartile 2	Quartile 3	Quartile 4	
	13.6% (8.1–15.5%)	17.3% (15.6–18.7)	20.0% (18.7–21.7)	25.2% (21.8–49.3)	
Hospitalizations / 100 beneficiaries	8.7	9.0	8.9	8.6	0.47
Annual ICU bed occupancy, %, Mean (range)	63.7% (33.1–95.9%)	66.1% (34.4–93.9)	63.2% (35.5–91.2)	64.1% (30.8–100%)	0.73
ICU beds, per 10,000 population, Median (range)	1.99 (0.79–4.47)	2.44 (0.90–5.05)	2.68 (1.14–9.20)	2.95 (0.83–10.50)	<0.01
Long-term acute-care beds, per 1,000 population, Median (range)	0 (0–50.6)	2.97 (0–78.9)	4.26 (0–44.2)	6.88 (0–58.6)	0.24
Skilled nursing beds, per 10,000 population, Median (range)	46.0 (11.5–129.3)	55.6 (16.5–173.8)	66.3 (21.4–132.9)	61.5 (13.9–144.3)	0.47
Intensivists / 100,000 population, median (range)	1.45 (0.4–3.28)	1.44 (0.21–3.01)	1.37 (0.25–3.81)	1.31 (0.2–2.95)	0.15
Specialists / 100,000 population, median (range)	122.1 (88.1–185.9)	118.6 (83.4–179.8)	115.0 (90.2–194.7)	114.1 (68.3–215.0)	0.08

ICU, intensive care unit; HRR, health referral region.

### Relationship between ICU admission rate and mortality—Bivariate analysis

**Fig 2:**
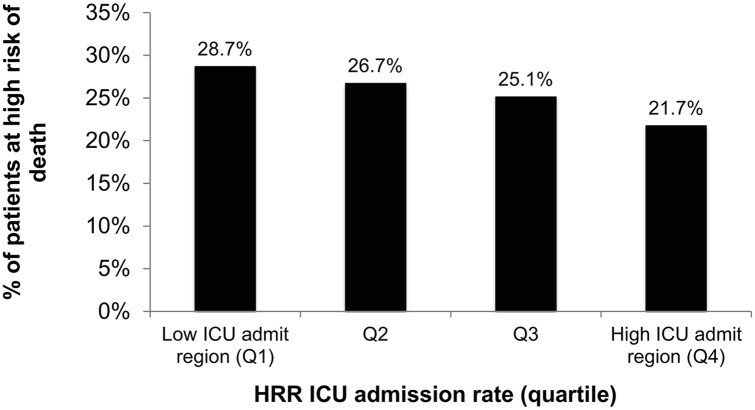
Fraction of patients at high-risk of death by strata of age-, sex-, race-adjusted HRR-level ICU admission. High risk of death defined as individuals in the top quartile of predicted risk of death.

Discrimination of the predictive model for 30-day mortality was good (AUC 0.79, S2 Table). Calibration was imperfect (Hosmer-Lemeshow p<0.01). After grouping individuals admitted to the ICU into quartiles of risk of death, the median risk of death at 30 days for mortality quartile 1 through 4 was 2.7%, 5.8%, 10.6%, and 28.8%, respectively. Patients with the greatest risk of death were more represented among HRRs in the lowest quartile of adjusted ICU admission rate (Fig 2). For example, only 21.7% of patients in HRRs in the highest quartile of ICU use had the greatest risk of death compared to 28.7% of patients in the lowest quartile of ICU use (p<0.001).

### Multivariable analysis

**Fig 3:**
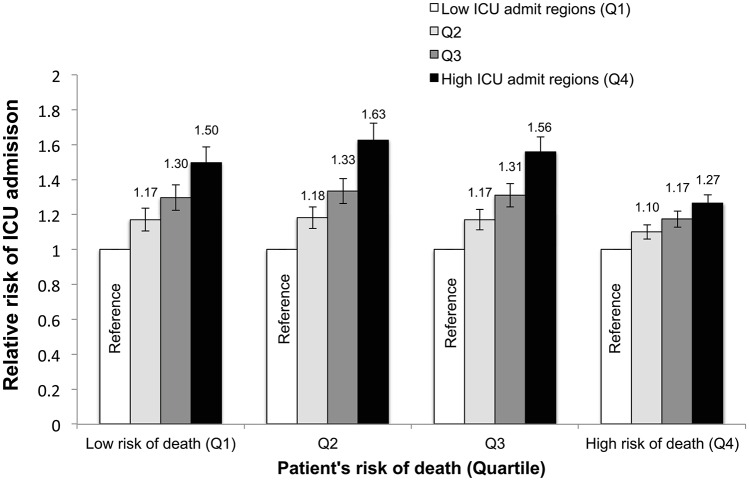
Association between regional ICU admission rate and patient-level risk of ICU admission, stratified by predicted risk of death. Relative risk represents the risk of ICU admission for a patient living in a given region relative to a region with a low ICU admission rate.

After adjusting for aggregate patient characteristics in the region and regional characteristics, individuals living in HRRs with greater ICU use were more likely to be admitted to the ICU regardless of their risk of death (Fig 3); however, risk of death modified this relationship (p for interaction <0.001). Among individuals with the greatest risk of death, living in a high ICU admission region (top HRR quartile) was associated with 27% increase in risk of ICU admission (RR 1.27, 95%CI 1.22–1.31), compared to the lowest quartile of ICU use. In contrast, for individuals with the lowest risk of death, living in high ICU admission region was associated with a 50% increase in the risk of ICU admission compared to the lowest quartile of ICU use (RR 1.50 95% CI 1.41–1.59). However, the greatest increase in likelihood of ICU admission across HRR quartiles was seen among individuals with moderate risks of death, where high compared to low ICU use regions increased risk of ICU admission by 63% and 56%, for patients in quartiles 2 and 3 for risk of death.

## Discussion

In this retrospective cohort study of patients hospitalized with one of a broad array of medical and surgical conditions, we demonstrated that there was wide variability in the use of the ICU across geographic regions, independent of measured patient characteristics including severity of illness, and regional characteristics. Patients with low, moderate, and high risks of death all contributed to regional variation in ICU admission rates. Region was a more important contributor to the likelihood of ICU admission for individuals with low risk of death compared to individuals with high risk of death. However, region was most important among individuals with moderate risks of death. These data suggest that variability in the use of the ICU is not limited to individuals with the greatest risk of death or those at the end of life, but is also attributable to individuals with low and moderate risk of death.

Although it may seem intuitive that geographic region is an influential driver of ICU admission for individuals with low to moderate risks of death, the empiric data to support this is limited. Most studies have focused on either quantifying hospital-level variation in use of the ICU for individuals at the end of life[21], or those with low risk conditions, such as diabetic ketoacidosis[22], congestive heart failure[23], acute myocardial infarction, pulmonary embolism[24], or describing geographic variation in use of the ICU for individuals at the end of life[25]. These studies suggest that ICU admission practice varies dramatically across hospitals regardless of the risk of death of the population studied, and across regions for individuals at the end of life. Our study complements this prior work and suggests that patients with low and moderate risks of death contribute to geographic variation in ICU use, and, in fact, are the exact population in which geographic region is most influential in determining ICU admission.

Our results have important implications for health system leaders and policy makers interested in improving the efficiency of ICU use. Much of the current focus on improving ICU efficiency revolves around eliminating overuse among individuals who are at the end of life. For example, a major thrust of the palliative care and hospice movements is to reduce unnecessary and often unwanted care during the dying process, including care provided in the ICU[26]. The focus of efforts of these movements is to encourage patients to articulate their values and goals of care and facilitating delivery of care concordant with those values and goals[27]. Though targeting a sizeable proportion of ICU use and total Medicare spending, these efforts may fail to maximize improvements in ICU efficiency[8, 28]. This is because they neglect individuals with lower risks of death who are also often unnecessarily admitted to the ICU and, as we have shown, strongly contribute to geographic variability in ICU admission rates.

The optimal way to reduce use of the ICU for individuals with little ability to benefit but are not at the end of life is unknown. One compelling proposal is through constraining ICU bed capacity to force providers to either implicitly or explicitly ration use of existing beds[29]. Several studies indicate that ICU bed availability is central to the decision to admit a patient to ICU, and providers often change their decision-making and goals of care conversations with patients when beds are scarce[30, 31]. Constraining ICU bed capacity could be accomplished through leveraging existing certificate of need laws in states where they currently regulate hospital beds. Recent data suggest that such efforts need not be national, but could target specific states where ICU bed capacity is rapidly growing[32]. However, it is important to note that the wide variability in ICU admission rates across regions that we observed was independent of measures of ICU, LTAC, and skill nursing bed capacity, suggesting that capacity constraints alone may be an inadequate remedy to inefficient use of ICU beds. Moreover, recent data suggests that capacity constraints, for some populations, may worsen care[33, 34]. Further research is needed to determine whether existing certificate of need laws effectively constrain ICU admission, and whether such constraints may identify a lower threshold in capacity beyond which patients may be harmed[33].

Alternatively, characterizing and targeting regional differences in provider norms of practice may also address the observed geographic variation in ICU use. Variation in provider norms of practice surrounding ICU triage may explain why a region’s influence on likelihood of ICU admission was greatest for individuals with moderate risk of death in our analysis. When providers confront ICU triage for this population—that is, individuals in the middle of the spectrum of disease severity and the greatest uncertainty surrounding their likelihood of benefit from the ICU—they may turn to heuristics, local norms, or protocols to aid in decision-making. For example, providers often rank ICU policies among the most important factors considered when making ICU triage decisions[30]. This is exemplified in recent work demonstrating that many hospitals require ICU admission for individuals with diabetic ketoacidosis requiring continuous insulin infusion[22, 35]. Uncertainty in a patient’s prognosis has also been linked to wide variability in resource use for the critically ill in other studies[36].

The extent to which these heuristics, norms, or protocols are variably employed across regions when there is uncertainty in a patient’s ability to benefit from ICU care is not known. Barnato and colleagues demonstrated that provider norms of care and staff perceptions of hospital norms at the end of life differ across geographic regions[37], but little is known about how such factors variably influence ICU triage for individuals with low or moderate risks of death. It is likely, given the greater uncertainty in outcomes and the ability to benefit from ICU care among individuals with moderate risk of death, that norms may vary dramatically, potentially contributing to differences in ICU triage. Future research charactering how norms of ICU triage, protocol use, and employment of heuristics when triaging a patient to the ICU is needed to identify alternative modifiable pathways to reduce overuse of the ICU.

Our study should be interpreted in the context of several limitations. The first is its use of administrative claims data and its inability to fully capture an individual’s risk of ICU admission and death. We estimated risk of death using the best available measures to account for severity of illness in administrate claims in a model with good discrimination, but there remains potential for such estimates to misclassify patients. The model’s calibration was imperfect, although this is often true of models built upon such large sample sizes[38]. Second, our study population included only individuals older than 65 years enrolled in fee-for-service Medicare. Although our results may not fully generalize to younger patients or those with other types of insurance, the majority of critical care in the United States is provided to individuals over age 65 years[39, 40]. Third, we were unable to account for regional variability in intermediate care ICU beds in our analysis, a growing alternative care location for individuals with critical illness, because no existing data source accurately captures these beds[41]. Finally, when characterizing the relationship between a region’s influence on risk of ICU admission and risk of death, we adjusted for several regional differences in population characteristics, bed supply, physician staffing, and market competition. Nevertheless, there remains potential for residual confounding from unmeasured regional characteristics.

## Conclusions

Care delivered in the ICU is an important contributor to growth in healthcare spending in the US and has become a focus of efforts to improve the value of inpatient care There is wide variation in in ICU use by geography, independent of ICU beds and physician supply, for patients with low and moderate risks of death. As survivorship of critical illness grows, efforts to improve ICU efficiency need to further delineate the causes of such geographic variation.

## Supporting Information

S1 Table International Classification of Disease, Ninth Revision, Clinical Modification (ICD-9-CM) Codes. (DOCX)

S2 Table Model for 30-day mortality (AUC = 0.79). (DOCX)

## References

[1] Wennberg JE, Fisher ES, Sharp SM, et al, for The Dartmouth Atlas of Health Care Working Group: *The Care of Patients with Severe Chronic Illness*: *An Online Report on the Medicare Program By the Dartouth Atlas Project*. Hanover, NH: Center for Evaluateive Clinical Sciences, Dartmouth Medical School, 2006 http://www.dartmouthatlas.org/atlases/atlas_series.shtm. Accessed June 1, 2010.

[2] Wennberg J, Gittelsohn. Small area variations in health care delivery. Science. 1973;182(117):1102–8. Epub 1973/12/14. 4750608 10.1126/science.182.4117.1102

[3] Wennberg JE, Freeman JL, Culp WJ. Are hospital services rationed in New Haven or over-utilised in Boston? Lancet. 1987;1(8543):1185–9. Epub 1987/05/23. 2883497 10.1016/s0140-6736(87)92152-0

[4] Seymour CW, Iwashyna TJ, Ehlenbach WJ, Wunsch H, Cooke CR. Hospital-level variation in the use of intensive care. Health Serv Res. 2012;47(5):2060–80. Epub 2012/09/19. 10.1111/j.1475-6773.2012.01402.x 22985033 PMC3513618

[5] Hogan C, Lunney J, Gabel J, Lynn J. Medicare beneficiaries' costs of care in the last year of life. Health Aff (Millwood). 2001;20(4):188–95. Epub 2001/07/21. 10.1377/hlthaff.20.4.188 11463076

[6] Luce JM, Rubenfeld GD. Can health care costs be reduced by limiting intensive care at the end of life? Am J Respir Crit Care Med. 2002;165(6):750–4. 10.1164/ajrccm.165.6.2109045 11897638

[7] Aronowitz RA, Asch DA. Cursing the darkness: are there limits to end-of-life research? J Gen Intern Med. 1998;13(7):495–6. Epub 1998/08/01. 10.1046/j.1525-1497.1998.00142.x 9686719 PMC1496984

[8] Barnato AE, McClellan MB, Kagay CR, Garber AM. Trends in inpatient treatment intensity among Medicare beneficiaries at the end of life. Health Serv Res. 2004;39(2):363–75. 10.1111/j.1475-6773.2004.00232.x 15032959 PMC1361012

[9] Chen LM, Render M, Sales A, Kennedy EH, Wiitala W, Hofer TP. Intensive care unit admitting patterns in the Veterans Affairs health care system. Arch Intern Med. 2012;172(16):1220–6. 10.1001/archinternmed.2012.2606 22825806

[10] The Dartmouth Atlas of Healthcare [October 12, 2015]. http://www.dartmouthatlas.org/tools/downloads.aspx.

[11] In: Newhouse JP, Garber AM, Graham RP, McCoy MA, Mancher M, Kibria A, editors. Variation in Health Care Spending: Target Decision Making, Not Geography. Washington (DC) 2013 10.17226/18393 24851301

[12] Seferian EG, Afessa B, Gajic O, Keegan MT, Hubmayr RD, Mayo E, et al Comparison of community and referral intensive care unit patients in a tertiary medical center: evidence for referral bias in the critically ill. Crit Care Med. 2008;36(10):2779–86. Epub 2008/10/02. 18828201 10.1097/ccm.0b013e318186ab1b

[13] Iwashyna TJ, Christie JD, Moody J, Kahn JM, Asch DA. The structure of critical care transfer networks. Med Care. 2009;47(7):787–93. Epub 2009/06/19. 10.1097/MLR.0b013e318197b1f5 19536030 PMC2760433

[14] Research Data Assistance Center. MEDPAR Intensive Care Unit (ICU) Indiator Code. http://www.resdac.org/cms-data/variables/MEDPAR-Intensive-Care-Unit-ICU-Indicator-Code Accessed October 12, 2015.

[15] Diez Roux AV, Merkin SS, Arnett D, Chambless L, Massing M, Nieto FJ, et al Neighborhood of residence and incidence of coronary heart disease. N Engl J Med. 2001;345(2):99–106. Epub 2001/07/14. 10.1056/NEJM200107123450205 11450679

[16] Birkmeyer NJ, Gu N, Baser O, Morris AM, Birkmeyer JD. Socioeconomic status and surgical mortality in the elderly. Med Care. 2008;46(9):893–9. 10.1097/MLR.0b013e31817925b0 18725842

[17] Elixhauser A, Steiner C, Harris DR, Coffey RM. Comorbidity measures for use with administrative data. Med Care. 1998;36(1):8–27. Epub 1998/02/07. 9431328 10.1097/00005650-199801000-00004

[18] Angus DC, Linde-Zwirble WT, Lidicker J, Clermont G, Carcillo J, Pinsky MR. Epidemiology of severe sepsis in the United States: analysis of incidence, outcome, and associated costs of care. Crit Care Med. 2001;29(7):1303–10. Epub 2001/07/11. 11445675 10.1097/00003246-200107000-00002

[19] Ehlenbach WJ, Hough CL, Crane PK, Haneuse SJ, Carson SS, Curtis JR, et al Association between acute care and critical illness hospitalization and cognitive function in older adults. JAMA: the journal of the American Medical Association. 2010;303(8):763–70. Epub 2010/02/25. 10.1001/jama.2010.167 20179286 PMC2943865

[20] Erickson GM, Finkler SA. Determinants of market share for a hospital's services. Med Care. 1985;23(8):1003–18. Epub 1985/08/01. 4021577 10.1097/00005650-198508000-00008

[21] Wennberg JE, Fisher ES, Stukel TA, Skinner JS, Sharp SM, Bronner KK. Use of hospitals, physician visits, and hospice care during last six months of life among cohorts loyal to highly respected hospitals in the United States. BMJ. 2004;328(7440):607 Epub 2004/03/17. 10.1136/bmj.328.7440.607 15016692 PMC381130

[22] Gershengorn HB, Iwashyna TJ, Cooke CR, Scales DC, Kahn JM, Wunsch H. Variation in use of intensive care for adults with diabetic ketoacidosis*. Crit Care Med. 2012;40(7):2009–15. Epub 2012/05/09. 10.1097/CCM.0b013e31824e9eae 22564962 PMC3561634

[23] Safavi KC, Dharmarajan K, Kim N, Strait KM, Li SX, Chen SI, et al Variation exists in rates of admission to intensive care units for heart failure patients across hospitals in the United States. Circulation. 2013;127(8):923–9. 10.1161/CIRCULATIONAHA.112.001088 23355624 PMC3688061

[24] Admon AJ, Seymour CW, Gershengorn HB, Wunsch H, Cooke CR. Hospital-level variation in ICU admission and critical care procedures for patients hospitalized for pulmonary embolism. Chest. 2014;146(6):1452–61. 10.1378/chest.14-0059 24992579 PMC4251611

[25] Fisher ES, Wennberg DE, Stukel TA, Gottlieb DJ, Lucas FL, Pinder EL. The implications of regional variations in Medicare spending. Part 1: the content, quality, and accessibility of care. Ann Intern Med. 2003;138(4):273–87. Epub 2003/02/15. 12585825 10.7326/0003-4819-138-4-200302180-00006

[26] Jenq G, Tinetti ME. Changes in end-of-life care over the past decade: more not better. JAMA. 2013;309(5):489–90. 10.1001/jama.2013.73 23385277

[27] Sudore RL, Fried TR. Redefining the "planning" in advance care planning: preparing for end-of-life decision making. Annals of internal medicine. 2010;153(4):256–61. Epub 2010/08/18. 10.7326/0003-4819-153-4-201008170-00008 20713793 PMC2935810

[28] Curtis JR, Engelberg RA, Bensink ME, Ramsey SD. End-of-life care in the intensive care unit: can we simultaneously increase quality and reduce costs? Am J Respir Crit Care Med. 2012;186(7):587–92. 10.1164/rccm.201206-1020CP 22859524 PMC3480521

[29] Gooch RA, Kahn JM. ICU Bed Supply, Utilization, and Health Care Spending: An Example of Demand Elasticity. JAMA. 2014. 10.1001/jama.2013.283800 24408679

[30] Escher M, Perneger TV, Chevrolet JC. National questionnaire survey on what influences doctors' decisions about admission to intensive care. BMJ. 2004;329(7463):425 Epub 2004/08/24. 10.1136/bmj.329.7463.425 15321898 PMC514202

[31] Stelfox HT, Hemmelgarn BR, Bagshaw SM, Gao S, Doig CJ, Nijssen-Jordan C, et al Intensive care unit bed availability and outcomes for hospitalized patients with sudden clinical deterioration. Archives of internal medicine. 2012;172(6):467–74. Epub 2012/03/14. 10.1001/archinternmed.2011.2315 22412076

[32] Wallace DJ, Angus DC, Seymour CW, Barnato AE, Kahn JM. Critical care bed growth in the United States. A comparison of regional and national trends. Am J Respir Crit Care Med. 2015;191(4):410–6. 10.1164/rccm.201409-1746OC 25522054 PMC4351597

[33] Hutchings A, Durand MA, Grieve R, Harrison D, Rowan K, Green J, et al Evaluation of modernisation of adult critical care services in England: time series and cost effectiveness analysis. BMJ. 2009;339:b4353 10.1136/bmj.b4353 19906740 PMC2776132

[34] Valley TS, Sjoding MW, Ryan AM, Iwashyna TJ, Cooke CR. Association of Intensive Care Unit Admission With Mortality Among Older Patients With Pneumonia. JAMA. 2015;314(12):1272–9. 10.1001/jama.2015.11068 26393850 PMC4758179

[35] Preeshagul I, Bajpayee G, Wunsch H, Cooke CR, Gershengorn HB. Understanding triage choices for patients with diabetic ketoacidosis admitted from emergency departments in New York. Journal of General Internal Medicine. 2014;29:S241–S.

[36] Detsky AS, Stricker SC, Mulley AG, Thibault GE. Prognosis, survival, and the expenditure of hospital resources for patients in an intensive-care unit. N Engl J Med. 1981;305(12):667–72. 10.1056/NEJM198109173051204 6790988

[37] Barnato AE, Tate JA, Rodriguez KL, Zickmund SL, Arnold RM. Norms of decision making in the ICU: a case study of two academic medical centers at the extremes of end-of-life treatment intensity. Intensive Care Med. 2012;38(11):1886–96. 10.1007/s00134-012-2661-6 22940755 PMC3684418

[38] Kramer AA, Zimmerman JE. Assessing the calibration of mortality benchmarks in critical care: The Hosmer-Lemeshow test revisited. Crit Care Med. 2007;35(9):2052–6. 10.1097/01.CCM.0000275267.64078.B0 17568333

[39] Angus DC, Kelley MA, Schmitz RJ, White A, Popovich J Jr. Caring for the critically ill patient. Current and projected workforce requirements for care of the critically ill and patients with pulmonary disease: can we meet the requirements of an aging population? Jama. 2000;284(21):2762–70. 11105183 10.1001/jama.284.21.2762

[40] Mullins PM, Goyal M, Pines JM. National growth in intensive care unit admissions from emergency departments in the United States from 2002 to 2009. Acad Emerg Med. 2013;20(5):479–86. 10.1111/acem.12134 23672362

[41] Sjoding MW, Valley TS, Prescott HC, Wunsch H, Iwashyna TJ, Cooke CR. Rising Billing for Intermediate Intensive Care Among Hospitalized Medicare Beneficiaries Between 1996 and 2010. Am J Respir Crit Care Med. 2015. 10.1164/rccm.201506-1252OC PMC4731714 26372779

