# Supplementary material for: Risk of Death Influences Regional Variation in Intensive Care Unit Admission Rates among the Elderly in the United States
Source: PLoS One. 2016 Nov 29;11(11):e0166933. doi: 10.1371/journal.pone.0166933 (PMC5127515; doi:10.1371/journal.pone.0166933)
Supplement: S1 Table — (DOCX) [file pone.0166933.s001.docx]

**S1 Table: International Classification of Disease, Ninth Revision, Clinical Modification (ICD-9-CM) Codes**

| **Diagnosis** | **ICD-9-CM Codes** | **Estimated discharges in 2011 Medicare (DRG)** | **Rank of all DRG by total discharges** |
| --- | --- | --- | --- |
| Acute myocardial infarction | 410.00 410.01 410.10 410.11 410.20 410.21 410.30 410.31 410.40 410.41 410.50 410.51 410.60 410.61 410.70 410.71 410.80 410.81 410.90 410.91 | 65,503 (280) | 34 |
| Congestive heart failure | 398.91, 404.X1, 404.X3, 428.0–428.9 | 223,501 (292) | 4 |
| Pneumonia | 480.X 481 482.XX 483.X 485 486 487.0 | 199,662 (194) | 6 |
| Chronic obstructive pulmonary disease | 490 4910 4911 4912 49120 49121 49122 4918 4919 4920 4928 494 4940 4941 496 | 303,488 (190,191) | 3 |
| Renal Failure | 584.5 584.6 584.7 584.8 584.9 586 | 154,063 (683) | 9 |
| Gastrointestinal hemorrhage | 456.0 4562.0 530.7 530.82 531.00 531.01 531.20 531.21 531.40 531.41 531.60 531.61 532.00 532.01 532.20 532.21 532.40 532.41 532.60 532.61 533.00 533.01 533.20 533.21 533.40 533.41 533.60 533.61 534.00 534.01 534.20 534.21 534.40 534.41 534.60 534.61 569.3 578.0 578.1 578.9 | 141,873 (378) | 14 |
| Ischemic stroke | 433.X1 434.X1 436 | 110,983 (065) | 21 |
| Colectomy | 45.73 - 45.76 or 17.31 17.32 17.33 17.34 17.35 17.36 17.39 45.8 45.81 45.82 45.83 | 56,994 (330) | 43 |
| Hip fracture | 81.51-81.55 or 7855 7905 7915 7925 7935 7965) AND primary diagnosis code of 820* | 428,495 (470) | 1 |
| Non-cervical spine fusion | 8100 8104 8105 8106 8107 8108 8109 8130 8134 8135 8136 8137 8138 8139 | 68,725 (460) | 36 |
| DRG, Diagnostic related group; ICD-9-CM, International classification of diseases, 9^th^ edition, clinical modification, source https://www.cms.gov/Research-Statistics-Data-and-Systems/Statistics-Trends-and-Reports/Medicare-Provider-Charge-Data/Inpatient2011.html | | | |
