# Supplementary material for: Risk of Death Influences Regional Variation in Intensive Care Unit Admission Rates among the Elderly in the United States
Source: PLoS One. 2016 Nov 29;11(11):e0166933. doi: 10.1371/journal.pone.0166933 (PMC5127515; doi:10.1371/journal.pone.0166933)
Supplement: S2 Table — (DOCX) [file pone.0166933.s002.docx]

**S2 Table – Model for 30-day mortality (AUC = 0.79)**

| **Variable** | | **Coefficient** | **Standard Error** | **P value** | **Definition** |
| --- | --- | --- | --- | --- | --- |
|  |  |  |  |  |  |
| **Intercept** | | -4.2015 | 0.0776 | <.0001 |  |
| **Age (years)** | |  |  |  |  |
|  | 70-74 | 0.1498 | 0.00966 | <.0001 |  |
|  | 75-79 | 0.3986 | 0.00891 | <.0001 |  |
|  | 80-84 | 0.6842 | 0.00861 | <.0001 |  |
|  | 85+ | 1.2156 | 0.00826 | <.0001 |  |
| **Female** | | -0.0978 | 0.00448 | <.0001 |  |
| **Black** | | -0.2902 | 0.00808 | <.0001 |  |
| **Socioeconomic category** | |  |  |  |  |
|  | 1 | 0.0802 | 0.0064 | <.0001 |  |
|  | 2 | 0.0678 | 0.00507 | <.0001 |  |
|  | 3 | 0 | . | . |  |
| **Elixhauser comorbidity** | |  |  |  |  |
|  | CHF | 0.3958 | 0.00582 | <.0001 |  |
|  | Valvular disease | -0.1721 | 0.0094 | <.0001 |  |
|  | Pulmonary Circulation Disorders | 0.2237 | 0.0183 | <.0001 |  |
|  | Peripheral Vascular Disorders | 0.1108 | 0.00874 | <.0001 |  |
|  | Paralysis | 0.4201 | 0.0163 | <.0001 |  |
|  | Other neurologic disorders | 0.4265 | 0.00742 | <.0001 |  |
|  | Chronic Pulmonary Disease | -0.00949 | 0.00504 | 0.0598 |  |
|  | Uncomplicated Diabetes | -0.0382 | 0.00561 | <.0001 |  |
|  | Complicated Diabetes | -0.1386 | 0.0114 | <.0001 |  |
|  | Hypothyroidism | -0.0883 | 0.00747 | <.0001 |  |
|  | Renal Failure | 0.6251 | 0.00818 | <.0001 |  |
|  | Liver Disease | 0.6462 | 0.0189 | <.0001 |  |
|  | Peptic Ulcer Disease | -0.2642 | 0.1163 | 0.0231 |  |
|  | AIDS | 0.053 | 0.1948 | 0.7857 |  |
|  | Lymphoma | 0.5454 | 0.0189 | <.0001 |  |
|  | Metastatic Cancer | 1.5407 | 0.011 | <.0001 |  |
|  | Solid Tumor | 0.7802 | 0.0113 | <.0001 |  |
|  | Collagen Vascular Disease | 0.0253 | 0.0158 | 0.1099 |  |
|  | Coagulopathy | 0.0469 | 0.0111 | <.0001 |  |
|  | Obesity | -0.3638 | 0.0193 | <.0001 |  |
|  | Weight Loss | 0.6367 | 0.00954 | <.0001 |  |
|  | Fluid and Electrolyte Disorders | 0.3946 | 0.00481 | <.0001 |  |
|  | Blood Loss Anemia | -0.2502 | 0.0134 | <.0001 |  |
|  | Deficiency Anemias | -0.113 | 0.00617 | <.0001 |  |
|  | Alcohol Abuse | -0.103 | 0.0214 | <.0001 |  |
|  | Drug Abuse | -0.3897 | 0.0621 | <.0001 |  |
|  | Psychoses | 0.0502 | 0.0167 | 0.0027 |  |
|  | Depression | -0.0072 | 0.0101 | 0.4739 |  |
|  | Hypertension | -0.3733 | 0.00471 | <.0001 |  |
| **Diagnoses present during admission** | | |  |  |  |
|  | Shock, non-trauma | 1.1247 | 0.0151 | <.0001 | Any diagnosis code 785.5X |
|  | Shock, trauma | 1.5813 | 0.3164 | <.0001 | Any diagnosis code 958.4 |
|  | Shock, post-op | -0.0821 | 0.0194 | <.0001 | Any diagnosis code 998 |
|  | Acute respiratory failure | 0.8942 | 0.00864 | <.0001 | Any diagnosis code 518.81 or 518.82 or 518.84 |
|  | Other pulmonary insufficiency | -0.0184 | 0.0246 | 0.4547 | Any diagnosis code 518.82 |
|  | Hypotension | 0.054 | 0.0116 | <.0001 | Any diagnosis code 458 |
|  | Respiratory arrest | 1.7575 | 0.0465 | <.0001 | Any diagnosis code 799.1 |
|  | Cardiac arrest | 2.2757 | 0.0193 | <.0001 | Any diagnosis code 427.5 |
|  | Acute myocardial infarction | 0.6687 | 0.0126 | <.0001 | Any diagnosis code 410.X (excluding 410.x2) |
|  | Infection | 0.367 | 0.00639 | <.0001 |  |
|  | Organ dysfunction (per Angus, et al.) | 0.6996 | 0.00908 | <.0001 |  |
|  | Explicit sepsis code (995.92,785.52) | 0.6227 | 0.0212 | <.0001 |  |
|  | Sepsis (Angus, et al) | -0.2926 | 0.00956 | <.0001 |  |
|  | CPR | 1.3247 | 0.0162 | <.0001 | Any procedure code 99.6 |
|  | Mechanical ventilation | 0.2713 | 0.0114 | <.0001 | Any procedure code 96.7X |
|  | Closed chest cardiac massage | 1.4893 | 0.1786 | <.0001 | Any procedure code 99.63 |
|  | Percutaneous coronary intervention | -1.1427 | 0.0165 | <.0001 | Any procedure code 36.01, 36.02, 36.05, 36.06, 36.09 |
|  | Valvular surgery | 0.0415 | 0.0511 | 0.4168 | Any procedure code 35.00-35.04, 35.10-35.14, 35.20-35.28, 35.96, 35.99 |
|  | CABG | -1.1644 | 0.0262 | <.0001 | Any procedure code 36.10 - 36.17, 36.19 |
| **Primary Diagnosis** | |  |  |  | See supplemental Table 1 |
|  | AMI | 0.6281 | 0.0782 | <.0001 |  |
|  | CHF | 0.8238 | 0.0771 | <.0001 |  |
|  | Pneumonia | 0.5979 | 0.0772 | <.0001 |  |
|  | COPD | 0.3268 | 0.0774 | <.0001 |  |
|  | Acute renal failure | 0.5933 | 0.0776 | <.0001 |  |
|  | Gastrointestinal hemorrhage | 0.5614 | 0.0774 | <.0001 |  |
|  | Ischemic stroke | 1.514 | 0.0773 | <.0001 |  |
|  | Colorectal surgery | -0.185 | 0.0758 | 0.0147 |  |
|  | Hip fracture surgery | 0.2777 | 0.0776 | 0.0003 |  |
|  | Non-cervical spine fusion | -1.15 | 0.0973 | <.0001 |  |
